# Impact of Povidone Application to Nares in Addition to Chlorhexidine Bath in Critically Ill Patients on Nosocomial Bacteremia and Central Line Blood Stream Infection

**DOI:** 10.3390/jcm13092647

**Published:** 2024-04-30

**Authors:** Raquel Nahra, Shahrzad Darvish, Snehal Gandhi, Suzanne Gould, Diane Floyd, Kathy Devine, Henry Fraimow, John E. Dibato, Jean-Sebastien Rachoin

**Affiliations:** 1 Division of Infectious Diseases, Department of Medicine, Cooper University Healthcare, Camden, NJ 08103, USA; fraimow-henry@cooperhealth.edu; 2 Division of Critical Care Medicine, Department of Medicine, Cooper University Healthcare, Camden, NJ 08103, USA; 3 Cooper Medical School of Rowan University, Camden, NJ 08103, USA; gandhi-snehal@cooperhealth.edu (S.G.);; 4 Department of Infection Prevention, Cooper University Healthcare, Camden, NJ 08103, USA; darvish-shahrzad@cooperhealth.edu; 5 Division of Hospital Medicine, Department of Medicine, Cooper University Healthcare, Camden, NJ 08103, USA; 6 Cooper University Healthcare, Camden, NJ 08103, USA; 7 Department of Nursing, Cooper University Healthcare, Camden, NJ 08103, USA

**Keywords:** nosocomial MRSA bacteremia, CLABSI, povidone-iodine, MRSA, decolonization, CHG

## Abstract

Nosocomial Methicillin-resistant Staphylococcus aureus (MRSA) bacteremia results in a significant increase in morbidity and mortality in hospitalized patients. We aimed to analyze the impact of applying 10% povidone iodine (PI) twice daily to both nares in addition to chlorhexidine (CHG) bathing on nosocomial (MRSA) bacteremia in critically ill patients. A quality improvement study was completed with pre and post-design. The study period was from January 2018 until February 2020 and February 2021 and June 2021. The control period (from January 2018 to May 2019) consisted of CHG bathing alone, and in the intervention period, we added 10% PI to the nares of critically ill patients. Our primary outcome is rates of nosocomial MRSA bacteremia, and our secondary outcome is central line associated blood stream infection (CLABSI) and potential cost savings. There were no significant differences in rates of MRSA bacteremia in critically ill patients. Nosocomial MRSA bacteremia was significantly lower during the intervention period on medical/surgical areas (MSA). CLABSIs were significantly lower during the intervention period in critically ill patients. There were no Staphylococcus aureus CLABSIs in critical care area (CCA)during the intervention period. The intervention showed potential significant cost savings. The application of 10% povidone iodine twice a day in addition to CHG bathing resulted in a significant decrease in CLABSIs in critically ill patients and a reduction in nosocomial MRSA in the non-intervention areas. Further trials are needed to tease out individual patients who will benefit from the intervention.

## 1. Introduction

Methicillin-resistant Staphylococcus aureus (MRSA) has been implicated in major nosocomial infections resulting in a significant increase in mortality, morbidity, length of stay, and direct healthcare costs [1,2,3,4]. Consequently, extensive research has focused on reducing the adverse effects on patients in hospitals. Implementing universal decolonization strategies in the critical care unit has been linked to a decrease in MRSA in clinical isolates including blood isolates [5,6] and has also been shown to be more cost-effective than targeted decolonization or standard of care (consisting of screening and isolation) [7].

Historically, MRSA decolonization strategies have typically involved the use of mupirocin for nasal application and the application of chlorhexidine (CHG) through wipes or bathing [7,8]. In several studies, the use of mupirocin and CHG for decolonization significantly reduced MRSA infections, though not all research found consistent results [9,10,11]. Additionally, the expense of mupirocin and the risk of developing resistance complicate its widespread application.

Povidone-iodine (PI) has been explored as an alternative to mupirocin for MRSA decolonization efforts [12,13]. In a randomized controlled trial focusing on surgical patients, nasal application of PI showed greater effectiveness in preventing Staphylococcus aureus infections at deep surgical sites compared to mupirocin [14]. In a recent study conducted within a nursing home setting, using four swabs of 5% PI and two swabs of 10% PI demonstrated similar efficacy in reducing MRSA colonization [13,15]. Additionally, PI has been documented to effectively decolonize MRSA in patients prior to undergoing surgery, further supporting its use as a pre-operative measure [16,17,18].

The Quality Improvement (QI) project focused on evaluating the effects of MRSA decolonization in patients within critical care units, specifically its influence on the occurrences of hospital-acquired MRSA bacteremia and central line-associated blood stream infection (CLABSI) within the critical care area (CCA) where the decolonization was implemented, as well as its impact on hospital-acquired MRSA bacteremia and CLABSI in the medical–surgical areas (MSA) where the intervention was not performed.

## 2. Methods

### 2.1. Study Design

The hospital where the study took place is a level 1 trauma, tertiary care center with 600 beds located in an urban area, on the east coast of the United States. The hospital services a very diverse population.

We conducted the QI project with a pre and post design using a time-based comparison of the effect of adding PI to nares to the usual practice of CHG bathing alone in adult critically ill patients. The QI was conducted in the CCA defined as medical ICU, trauma ICU, cardiac ICU and neuro ICU. We excluded the neonatal ICU (NICU) patients from the intervention.

The institutional IRB deemed the study exempt from full IRB review.

### 2.2. Intervention

During the control period, all patients received daily baths with 2% CHG wipes.

During the intervention period, in addition to the CHG wipes, every patient admitted to a CCA had each nostril swabbed with 10% PI twice a day starting on the morning of the second calendar day of admission to a CCA. This continued until the patient’s discharge from the CCA.

The intervention period spanned from May 2019 to February 2020 and then from February 2021 to June 2021, making it a cumulative duration of 20 months. The project was temporarily suspended due to the COVID-19 pandemic, which necessitated alterations in our established infection prevention protocols due to shortages of supplies and the adoption of crisis standards of care. The intermittent availability of PI, personal protective equipment (PPE), and cleaning supplies, coupled with uncertainties about potential future outbreaks, influenced our decision to pause the project temporarily. The project resumed once vaccine distribution began, and the supply chain showed signs of stabilization. The control period, lasting 16 months from January 2018 to April 2019, included the time immediately before the start of the intervention.

### 2.3. Education and Quality Assessment

Nursing education on the use of 10% PI consisted of sharing information on multiple huddles during the day and night shifts as well as distribution of written instructions on how to use the PI. Nurses were also instructed on how to document the use of the PI in a flowchart in the electronic medical records (EMRs). The education was a combined ongoing effort between unit leaders and nurse educators in the target units to ensure quality and compliance. 

We monitored the weekly utilization of 10% PI as part of our process for replenishing supplies to the various units. Additionally, we extracted data on PI utilization from the EMR. There were no changes in the monitoring of Environmental Services (EVS), hand hygiene procedures, or products during the intervention period.

### 2.4. Definitions

Nosocomial or healthcare-associated MRSA bacteremia is defined by the National Healthcare Safety Network (NHSN) as new MRSA bacteremia that developed on day 4 of hospitalization [19]. In clinical laboratories, any Staphylococcus aureus strain exhibiting an oxacillin MIC greater than 4 is automatically entered into the NHSN database, where it is subsequently reported as a standardized infection ratio (SIR) [20]. In this QI, we will report this variable as a rate. The denominator is patient days for the specific unit to which the nosocomial MRSA bacteremia is attributed to.

CLABSI per NHSN is a bacteremia associated with a central or peripherally inserted central catheter, not attributed to a secondary site or meeting criteria for a commensal organism. For purpose of this QI, we are also reporting this variable as a rate. The denominator is line days.

Since nosocomial methicillin-sensitive Staphylococcus aureus (MSSA) and Gram-negative rod (GNR) are not tracked by NHSN, we used CLABSI involving MSSA and GNR as a surrogate for hospital-onset bacteremia [21]. Details of organisms associated with CLABSI during the study period and designated comparative period is in Table 1. Note that we combined under Enterobactereales the following organisms: *Escherichia coli*, *Klebsiella pneumonia*, *Proteus mirabilis*, *Morganella morganii*, and *Serratia marcescens*. Some CLABSI were polymicrobial accounting for a higher number of microorganisms isolated compared to CLABSI.

### 2.5. Outcome Measure

The main focus of the study was to determine the rate of nosocomial MRSA bacteremia in the Critical Care Area (CCA). Secondary endpoints encompassed the rate of nosocomial MRSA bacteremia in the medical–surgical area (MSA), along with the rate of CLABSI in both CCA and MSA. Additionally, the study aimed to identify the discrete number of bacteremia cases and the specific organisms involved in CLABSI. All data were collected by the quality team and infection preventionists in accordance with the Centers for Medicare & Medicaid Services (CMS) requirements for reporting [22,23]. We analyzed the data gathered during the QI initiative and the pre-identified comparison period. Individual patient information was not collected, as it was outside the scope of this study. The cost of care analysis was conducted using extrapolated data from the Agency for Healthcare Research and Quality (AHRQ), along with the number of MRSA bacteremia and CLABSI cases. To maintain accuracy, the cost analysis relied on AHRQ published data, preventing cost inflation in the analysis of events among critically ill patients.

### 2.6. Statistical Analysis

We present the rates of infections as N/1000-line days for CLABSI and N/1000 patient days for nosocomial MRSA in blood culture as the mean (95% CI). The trend in the use of PI was explored. We performed a non-parametric Kernel regression analysis to determine whether universal decolonization (use of PI) was associated with nosocomial MRSA and CLABSI. Differences in the number of microorganisms associated with CLABSI before and after PI were also explored using the Wilcoxon signed-rank test. All analyses were conducted in R version 4.2.2 and conclusions made at 5% significance level.

## 3. Results

During the intervention period, the rate of nosocomial MRSA in blood cultures was significantly lower in MSA. However, in CCA, although there was a decrease in the total number of nosocomial MRSA cases in blood cultures, this reduction did not reach statistical significance (Table 2).

**Figure 1:**
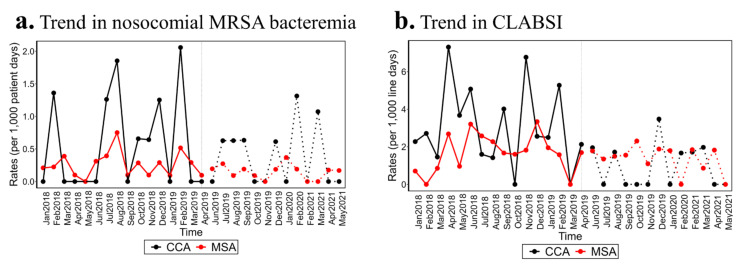
Nosocomial MRSA bacteremia and CLABSI rates in CCA and MSA January 2018–June 2021. (**a**): Temporal trends in the number of nosocomial MRSA bacteremia per 1000 patient days before and after the intervention periods. (**b**): Temporal trends in the number of CLABSI per 1000 line days before and after the intervention periods. (**a**,**b**) are the raw counts divided by the patient days or line days for each month.

In the critical care area (CCA), the rate of CLABSI was significantly lower during the intervention period (1.05 ± 1.05) compared to the control period (2.7 ± 2 CLABSI/1000-line days). However, there was no difference in CLABSI rates in the medical–surgical area (MSA) (see Figure 1). Upon closer examination of the microbiology results from the CLABSI cases, it was observed that there were no instances of Staphylococcus aureus CLABSI during the intervention periods in the CCA (see Table 1).

Using CLABSI as a surrogate marker for bacteremia, the intervention led to a significant reduction in bacteremia cases across both the CCA (*p*-value = 0.0068, see Table 1) and the combined CCA+MSA (*p*-value = 0.0313). While there was a decrease in the incidence of bacteremia in the non-intervention area (MSA) during the QI period, this reduction did not reach statistical significance.

**Figure 2:**
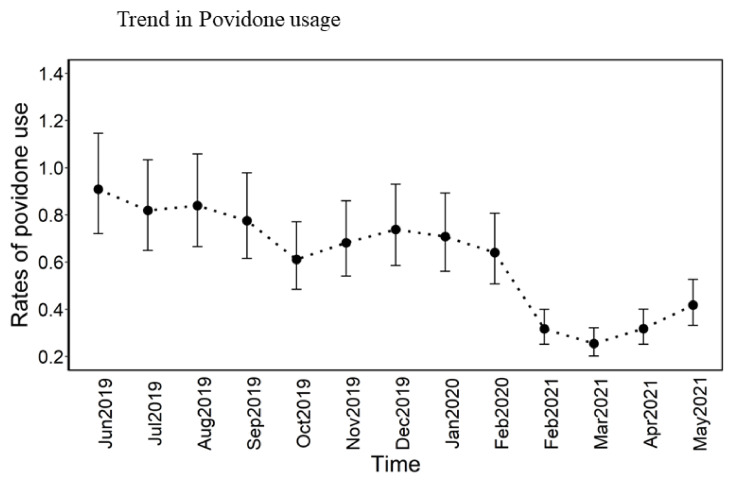
Povidone Iodine usage in CCA: Temporal trends in the usage of povidone iodine adjusted for length of stay for each month.

Despite consistent use of PI between June 2019 and February 2020, there was a significant drop in usage after February 2020 (see Figure 2).

Although the PI swabs were provided free of charge, we estimated the cost of the intervention based on the usage of swabs during the intervention period. Swabbing patients’ nostrils twice a day was not labor-intensive and did not require a higher level of skill from the nursing staff, so no additional cost was associated with this task. Therefore, the estimated cost of the intervention is based solely on the actual cost of the swabs. The estimated total cost for supplies, based on the 2022 market price, is USD 53,500. AHQR estimates the cost of nosocomial MRSA bacteremia to be between USD 32,000 and USD 36,000 [24]. The reduction of 20 nosocomial MRSA bacteremia events could result in an estimated average cost saving of USD 34,000 per incident, leading to a potential total cost saving range of USD 640,000 to USD 720,000 for the institution. This calculation assumes that none of the nosocomial MRSA bacteremia cases are associated with ventilator-associated pneumonia (VAP), as the cost of pneumonia is higher than that of septicemia alone, even when adjusted for length of stay. The cost of CLABSI according to AHQR in 2017 is estimated to be USD 48,000, with a range of USD 27,000 to USD 69,000 [25]. During the intervention period, there were 21 fewer incidents of CLABSI throughout the institution. Therefore, the savings from using PI could be estimated at USD 619,000 to USD 703,000 for CLABSI alone. The overall predicted cost savings, factoring in product cost and savings on CLABSI and nosocomial MRSA bacteremia, amounted to USD 2,005,850.

## 4. Discussion

In this project, we observed that combining PI swabs to nares with CHG application on the skin significantly decreased CLABSI incidence among patients in critical care. This intervention also showed potential for substantial cost savings. Interestingly, while there was no significant decrease in nosocomial MRSA bacteremia in the target units during the intervention period, we did not observe any cases of Staphylococcus aureus CLABSI in the Critical Care Unit during the same period. These results are consistent with findings in a similar intervention in the UK [9].

Regrettably, our QI coincided with the onset of the COVID-19 pandemic. The pandemic placed significant strain on our nursing staff. Several studies have established that quality measures, including healthcare-associated infections, are profoundly influenced by the experience and ratio of nursing staff [26,27]. The nursing staff shortage resulting from illness, staff seeking travel opportunities, and new nurses entering the workforce, coupled with increased patient acuity and supply chain challenges, has negatively impacted the quality of care delivered [28,29,30]. In our institution, an analysis of EMR aggregate data revealed a significant decrease in our usage of PI swabs to the nares after February 2020 (see Figure 2). It is important to highlight that the periods during which nosocomial MRSA bacteremia occurred overlapped with a reduced compliance with the intervention (see Figure 1). Regrettably, we were unable to control for or incorporate the impact of COVID-19 on the quality of care delivered in our statistical analysis. We acknowledge that this is not a unique issue to our institution [30,31,32]. With a particular focus on hospital-acquired infections (HAI), the NHSN report published in 2022 shows a significant increase in all HAI tracked by NHSN in 2020 compared to 2019 [33]. For instance, 2020 CLABSI SIR started with a 12% decrease compared to the same period in 2019; however, as COVID-19 settled, a significant increase was seen. In the state where the QI was carried out, the 2020 third quarter (Q3) CLABSI SIR of 0.86 was 59% higher than the SIR from 2019-Q3 of 0.54. CLABSI and nosocomial MRSA bacteremia also known as MRSA lab ID are considered quality metrics. It is worth mentioning that patients with COVID-19 infection are noted to be at an increased incidence of Staphylococcus aureus infection [33,34,35]. Despite a nationwide increase in MRSA bacteremia, our institution’s simple intervention, which involved applying PI swabs to the nares while continuing to use CHG wipes for critically ill patients, helped us to keep the number of infections below pre-pandemic levels in the CCA. Although this outcome did not reach statistical significance, we did not experience a similar increase in MRSA bacteremia (99% increase) as observed in other institutions in our state.

PI swabs application in addition to CHG resulted in a significant decrease in CLABSI from all causes and no Staphylococcus aureus CLABSI. With CLABSI being used as a surrogate for healthcare-associated bacteremia [21], the intervention had a beneficial effect on overall healthcare-associated bacteremia in the CCA, though we did not evaluate if that translated in improved patient outcome [36] as the study was not designed to assess individual patients. An additional drawback was our inability to assess real-time PI swab use or intervene if PI swabs were not used for each patient; we extrapolated compliance based on distribution to units and did education if usage went down.

Moreover, the project was a quality improvement project and did not have the rigor of randomized control trials. Our approach, as any QI, has the benefit of mimicking real-life situations [37]. As described above, the cost of the intervention would have been based on the cost of PI swabs to nares, which would have been USD 53500 for the duration of the QI. Given the structure of QI projects, we pulled the nosocomial MRSA bacteremia from NHSN which allows for duplicate events if the inpatient location of the patient is different when the initial blood culture is obtained. This inability to remove duplicates might inflate our nosocomial MRSA bacteremia rates. Therefore, a more structured study design such as a randomized controlled trial that is appropriately powered might be able to answer questions regarding the patient population where the intervention would have the highest impact.

## 5. Conclusions

Application of PI swabs twice a day in addition to CHG bathing resulted in a significant decrease in catheter-associated blood stream infections in critically ill patients. The impact of the intervention on nosocomial MRSA bacteremia and outcomes such as mortality and morbidity needs to be further evaluated in a randomized trial.

## Figures and Tables

**Table 1:** Microbiology results for CLABSI before and after intervention. This table displays the individual cases of central line-associated bloodstream infections (CLABSI) and the attributed microorganisms before and after the intervention was implemented in the CCA. Several CLABSI cases were polymicrobial, which accounted for the discrepancy between CLABSI cases and bacterial counts.

Bacteria	MSA Pre	MSA Post	MSA *p*-Value	CCA Pre	CCA Post	CCA *p*-Value
*Pseudomonas aeruginosa*	2	3		1	2	
*Stenotrophomonas maltophilia*	1	1				
*Escherichia coli*	1	3		2	0	
*Proteus mirabilis*	0	1				
*Morganella morganii*	1	0				
Coagulase-negative staphylococci	2	3		2	1	
*Streptococcus mitis-oralis*	0	0				
*Staphylococcus aureus*	9	6		5	0	
*Klebsiella oxytoca*	2	0				
*Klebsiella pneumoniae*	3	2		2	1	
*Serratia marcescens*	0	0		1	0	
*Citrobacter koseri*	0	0				
*Acinetobacter baumannii complex*	3	0		3	0	
*Candida* spp.	4	2		5	4	
*Enterococcus* spp.	4	1		4	0	
*Fusobacterium* spp.				1	0	
*Bacteroides fragilis*				2	0	
Total count	32	22	0.16	28	8	0.0068

**Table 2:** Rates of nosocomial MRSA bacteremia and CLABSI during the study period. The MRSA column represents the nosocomial MRSA bacteremia single events per unit within 14 days divided by patient days for the assigned area analyzed. CLABSI represents events of bacteremia associated with a central line divided by line days in the specific area.

	Nosocomial MRSA Bacteremia			CLABSI		
	Before	After	*p* Value	Before	After	*p* Value
CCA	0.57 (0.10, 4.38)	0.38 (0.05, 3.97)	0.4	3.05 (1.08, 8.13)	0.96 (0.18, 4.80)	0.0075
MSA	0.26 (0.02, 3.68)	0.14 (0.02, 3.68)	0.05	1.68 (0.35, 5.69)	1.37 (0.22, 5.26)	0.34
Total	0.41 (0.06, 4.04)	0.26 (0.03, 3.83)	0.22	2.37 (0.71, 6.91)	1.16 (0.19, 5.03)	0.06

## Data Availability

The raw data supporting the conclusion of this article and educational material for nursing staff will be made available by the authors on request.
